# Bidirectional promoters in seed development and related hormone/stress responses

**DOI:** 10.1186/1471-2229-13-187

**Published:** 2013-11-22

**Authors:** Sofia Kourmpetli, Kate Lee, Rachel Hemsley, Pascale Rossignol, Thaleia Papageorgiou, Sinéad Drea

**Affiliations:** 1 Department of Biology, University of Leicester, University Road, Leicester LE1 7RH, UK; 2 Bioinformatics and Biostatistics Analysis Support Hub (BBASH), College of Medicine, Biological Sciences and Psychology, University of Leicester, Leicester, UK; 3 Current address UCL Business PLC, The Network Building, 97 Tottenham Court Road, London W1T 4TP, UK; 4 Current address Department of Molecular, Cellular and Developmental Biology, Yale University, New Haven, CT 06520, USA

**Keywords:** Arabidopsis thaliana, Bidirectional promoter, G-box, ABRE, Abscisic acid, Seed development, Desiccation, Stress response

## Abstract

**Background:**

Bidirectional promoters are common in genomes but under-studied experimentally, particularly in plants. We describe a targeted identification and selection of a subset of putative bidirectional promoters to identify genes involved in seed development and to investigate possible coordinated responses of gene pairs to conditions important in seed maturation such as desiccation and ABA-regulation.

**Results:**

We combined a search for 100–600 bp intergenic regions in the Arabidopsis genome with a *cis*-element based selection for those containing multiple copies of the G-box motif, CACGTG. One of the putative bidirectional promoters identified also contained a CE3 coupling element 5 bp downstream of one G-box and is identical to that characterized previously in the *HVA1* promoter of barley. CE3 elements are significantly under-represented and under-studied in Arabidopsis. We further characterized the pair of genes associated with this promoter and uncovered roles for two small, previously uncharacterized, plant-specific proteins in Arabidopsis seed development and stress responses.

**Conclusions:**

Using bioinformatics we identified putative bidirectional promoters involved in seed development and analysed expression patterns for a pair of plant-specific genes in various tissues and in response to hormones/stress. We also present preliminary functional analysis of these genes that is suggestive of roles in seed development.

## Background

Bidirectional promoters are common in genomes [1] and have more recently been identified and examined *in silico* in the completed genomes of plants, including Arabidopsis [2,3]. The relevance and potential of these promoters in biotechnology has been documented [4-6], particularly for use in gene-stacking approaches where more than one gene is required to confer a particular trait trangenically or more than one trait is being conferred e.g. resistance to a suite of pests [7]. While researchers can engineer polar promoters to be bidirectional [4,6], if we can characterize naturally occurring bidirectional promoters in plants these could provide a valuable alternative or at least a source of information on their mode of action *in planta*.

As well as applied and biotechnological relevance, the existence of bidirectional promoters has been recognized as a fundamental and complex means of transcriptional control [8,9]. Research in yeast revealed that the existence of bidirectional promoters was not only pervasive but the source of the majority of cryptic transcription in the organism and therefore the means of transcriptional regulation [10,11].

Except for isolated examples of detailed experimental analyses of bidirectional promoters [12-15], virtually all the work published to date is bioinformatics-based and while this work has certainly highlighted the prevalence and potential importance of bidirectional promoters in areas from fundamental transcriptional control research to clinical relevance in cancer research [8,9,16], it still remains to explore the functional relevance of this gene organization experimentally by focusing on specific gene pairs of interest. The bioinformatics/computational-based criteria used to isolate a workable set of genes can vary depending on the desired outcome of the analyses and can involve coding and non-coding features – in this case we used both to identify putative bidirectional promoters regulating genes involved in seed development. Analysis of the divergently transcribed genes associated with a targeted set of bidirectional promoters is likely to lead to discovery of novel genes involved in development and/or previously undescribed relationships between genes of different functional categories in common or complementary processes/responses.

We used bioinformatics to search and identify a subset of putative bidirectional promoters that we predicted would regulate genes with roles in seed development. ABA is integral to plant seed development as a general process but mechanistically ABA mediates the conferral of desiccation tolerance and dormancy on seeds [17]. As such therefore there is extensive crosstalk between the stress responses of drought and cold as well as antagonistic interactions with the germination process [18]. The response to ABA is mediated by promoter motifs based on the ACGT core called ABREs (abscisic acid responsive elements) and including the G-box element (CACGTG). This element has been found in previously identified cases of bidirectional promoters which regulate genes involved in ABA response and seed development [19-21], as well as in promoters of genes regulated by light [22,23]. The identity of the nucleotides flanking the ACGT has been found to be an important determinant of the element’s specificity [24,25]. The use of *cis*-elements such as ABREs in identifying genes involved in ABA and stress response has been previously described [26]. The G-box ABRE is often found in combination with other motifs, coupling elements (CE) that can be derived from or distinct from the ACGT core and are also involved in the seed/ABA regulation and responses to osmotic and cold-temperature stresses [18,27]. In addition, specific motifs associated with regulation by cold and dehydration have been classified as DRE/CRTs (dehydration response element/c-repeat) derived from a CCGAC core sequence [28,29].

Previous work characterized two genes highly expressed in maturing seeds [30,31]. These genes are transcribed from an intergenic region (between start codons) of 411 bp which contains three copies of the G-box (CACGTG) motif involved in ABA-regulated seed development [20,30,31] (Additional file 1: Figure S1A). These genes, At4g16155 and At4g16160, encode a plastid outer envelope protein, OEP16-S, and a lipoamide dehydrogenase, ptLPD2, also localized to the plastid (in this case the stroma) [31]. Recent functional analyses of these genes revealed roles in metabolic fluctuation and arsenate sensitivity, respectively [32,33]. In addition, a divergent arrangement of a seed-expressed oleosin gene, *OLE1*, and a peptide methionine sulfoxide reductase, *PMSR*, At4g25130 and At4g25140 respectively, was reported initially in *Brassica napus* and then characterised in *Arabidopsis*[21,34,35]. This promoter of 499 bp (between start codons) contained two copies of the G-box motif (Additional file 1: Figure S1B). The PMSR protein is plastid-localised and involved in oxidative stress response while mutations in *OLE1* conferred cold tolerance [36,37].

We combined a search for a bidirectional gene arrangement with ABRE and associated *cis*-elements in the Arabidopsis genome and subsequently focused on a pair of plant-specific genes of unknown function that we characterised in detail. The identity and localisation of the genes identified in the bioinformatics search suggest that this gene arrangement might enable a means of concerted or complementary responses to stresses or environmental stimuli, such as drought or hormones, while the localization of the gene products to varied organelles could reflect a means of coordinating the complex intracellular interactions induced by stress conditions.

## Methods

### Bioinformatic analyses

*Arabidopsis thaliana* genes were downloaded from ENSEMBL plants 17, with headers including gene ID, transcript ID, coding sequence, chromosome name, transcript start, transcript end and strand. Putative bidirectional promoters were identified using in-house Perl scripts that searched header information for transcripts on opposite strands of the same chromosome that had start sites within 100–600 bp of one another. (The start sites of transcripts on the reverse strand were taken as ENSEMBL’s ‘transcript end’ position). Putative bi-directional promoter sequences were retrieved from ENSMBL plant 17 chromosomes and searched for the presence of the CACGTG motif.

We used the AtGenExpress Visualisation Tool (AVT; http://jsp.weigelworld.org/expviz/expviz.jsp) to extract gene expression data where indicated using the datasets for development, abiotic stress, hormones and light [38-40].

### Sequence analysis

Homologous sequences to genes At3g03150 and At3g03160 were identified using standard BLAST searching tools (http://www.ncbi.nlm.nih.gov/BLAST/). The Accession numbers of homologous sequences used for amino acid alignments are shown in Additional file 2: Table S1). Alignments were done using CLUSTALX and BioEdit [41,42]. Localisation prediction programs used were PSORT [43] and TargetP [44]. Searches for promoter motifs were done using PLACE [45] and transmembrane prediction was performed using the TMHMM Server v2.0 [46].

### Plant material and growth conditions

*Arabidopsis thaliana* wild-type (Col-0) and T-DNA insertion plants were grown in a control environment with a 16 h photoperiod, 120–140 μmol/m^2^/sec light intensity, 40-50% relative humidity and a temperature of 21 ± 2°C. For crosses with dehiscent anthers, closed flower buds were emasculated 48–72 h before pollination.

For silique analysis, the five longest green siliques were collected from each plant (wild-type, T-DNA insertion line or cross between the two), opened under a dissecting microscope and the number of normal seeds, early and late aborted seeds as well as unfertilized ovules was determined.

### Stress treatments

Arabidopsis seedlings were grown vertically for three weeks at 22°C on a medium containing 0.5xMS salts, 0.5xMS vitamins, 0.5 gl^-1^ 4-morpholineethanesulfonic acid (MES), 0.5% (w/v) sucrose and 0.7% agar, pH 5.7. For each treatment seedlings were transferred into a petri dish with a sterile filter paper that was soaked in either liquid MS medium, 10 μM ABA in 0.1% methanol or 0.1% methanol. Three seedlings from each treatment were collected for further analysis after 3 h and 24 h. For the dehydration stress, seedlings were left in an open petri dish at room temperature for 1.5 h. Primers for the *KIN1* and *RD29* genes were taken from Kim *et al.*[47].

### Protein localization

The full-length coding region of At3g03150 was amplified from the SALK ORF-trimmed pUNI clone (u23027) with Gateway compatible primers and cloned into a pDONR207 entry vector (Invitrogen). This was used to make a C-terminal GFP fusion in GFP-C-BIN and subsequent transient transformation of tobacco cells. Tobacco leaves (*Nicotiana benthamiana*) were infiltrated with a solution of saturated *Agrobacterium* resuspended in 10 mM MgCl_2_, 10 mM MES, 100 μM acetosyringone to OD_600_ 0.4) and observed for GFP localisation 10 days after infiltration. Mitochondria tracker CMX Ros red (Molecular Bioprobe) was used at 400 nM in water for 45 min and washed 3 times for 5 min in water. Observation was carried out on an inverted SP2 confocal (Leica) using a 40x oil immersion lens. Sequential scans were taken with GFP excited at 488 nm with an Argon Ion laser and the Mito tracker at 543 nm from a green helium neon laser.

### Transcript analysis by RT-PCR and qRT-PCR

Total RNA was extracted from the flower tissues of insertion lines and from flower, silique, root, rosette and cauline leaf and stem tissues of wild-type Arabidopsis and cDNA was made using the BioScript kit (Bioline) following the manufacturer’s instructions. Primers for amplification of At3g03150 from cDNA were 150 F and R; for At3g03160, 160 F and R; for At5g17165, 165 F and R and for At5g17190, 190 F and R. Actin was used as control using primers Actin2F and Actin2R.

Total RNA was extracted from ABA/stress treated seedlings of *A. thaliana* using TriSure™ (Bioline) and treated with DNase I (NEB), according to the manufacturer’s instructions. 700 ng of RNA from each sample were used in a 20 μl cDNA synthesis reaction with the Tetro cDNA Synthesis Kit (Bioline), following the manufacturer’s instructions. Quantification of At3g03150 and At3g03160 transcript levels by real-time PCR was performed using 1 ul of a 1:20 dilution of cDNA template in a 20 ul reaction containing SYBR Green JumpStart™ Taq ReadyMix™ (Sigma-Aldrich) and primers 150 qF and 150 qR, or 160 qF and 160 qR at a final concentration of 0.5 uM. Each reaction was performed in triplicate in a PTC-200 Peltier thermal cycler (MJ Research) using the following conditions: denaturation at 95°C for 3 min followed by 40 cycles of denaturation at 95°C for 30 sec, annealing at 55°C for 30 sec and extension at 72°C for 30 sec. 18S was used the reference gene with primers 18S F and 18S R All primers are listed in Additional file 3: Table S2.

### Promoter cloning

The At3g03150-At3g03160 promoter sequence (between the two ATG start codons) was amplified from Col-0 genomic DNA using primers AtPromF and AtPromR (Additional file 3: Table S2) and a proofreading DNA polymerase (Velocity, Bioline). The amplified genomic fragment was then cloned into pJET1.2 vector using the CloneJET PCR Cloning Kit (Thermo Scientific) and confirmed by sequencing. The promoter fragment was amplified from pJET1.2 vector in both orientations using primers with suitable attB sites attached to them and cloned into Gateway® entry vector pDONR221 (Invitrogen). The primers used were 150promF and R for At3g03150 160promF and R for At3g03160. Each promoter entry clone was introduced into pKGWFS7 destination vector using single site recombination, in order to access the ability of the intergenic region to drive GUS expression in both orientations.

### Plant transformation

Arabidopsis Col-0 plants were transformed with *Agrobacterium tumefaciens* GV3101 strain harboring either one of the two promoter-pKGWFS7 plasmids using a standard floral dipping method [48]. T1 seed collected from the transformed plants was plated on kanamycin selection plates. Surviving seedlings were transferred to soil and used for further analysis.

### GUS staining

Siliques were harvested at stages just after fertilisation and up to endosperm cellularistion, fixed in 90% acetone at −20°C, infiltrated (under vacuum for 1 mintue) with GUS staining solution (50 mM Na_2_HPO_4,_ 50 mM NaH_2_PO_4_, pH 7.0, 2 mM potassium ferricyanide, 2 mM potassium ferrocyanide, 2 mM EDTA, 1 mg/ml X-Gluc) and incubated at 37°C for overnight. The same staining solution was used to infiltrate fresh tissues of seedlings, leaves and inflorescences. After staining, tissue was cleared with 70% ethanol and stored at 4°C.

### Microscopy

GUS-stained ovules and ovules for phenotypic analyses were mounted in chloral hydrate and analysed with DIC optics as described in Boisnard-Lorig *et al.*, [49]. Images were captured using a digital camera and assembled with Adobe Photoshop software (Adobe Systems, Mountain View, CA).

### Insertion lines characterization

SALK T-DNA lines SALK_121507 and SALK_025090 [50] were obtained through NASC (Nottingham Arabidopsis Stock Centre) and genotyped by PCR as recommended using the LBb1.3 primer and gene specific primers 507 F and R; 262 F and R (Additional file 3: Table S2).

### Genetic transmission through male and female gametes

In order to determine the gametophytic transmission efficiency (TE) of the T-DNA, reciprocal crosses between wild-type Col-0 and SALK_121507 or SALK_025090 plants were performed. Seed was collected from individual siliques and the F1 generation was screened for the presence of the T-DNA insertion. The TE through each gamete (TE_MALE_ and TE_FEMALE_) was calculated according to Howden *et al.*[51].

### Pollen analyses

Pollen of wild-type and T-DNA insertion lines was stained with DAPI (4′,6-diamidino-2-phenylindole; 1 mg/ml) and examined for any morphological differences. FDA (fluorescein diacetate) staining was used to access viability (10% solution in acetone/0.3 M mannitol). Pollen germination assays were performed according to Boavida and McCormick [52].

## Results

### Identifying putative ABA-regulated/seed-expressed bidirectional promoters in Arabidopsis

We performed a promoter *cis*-element bioinformatics search based on specific examples of bidirectional gene pairs involved in aspects of seed biology [20,21,30,31,33,34,36]. We identified putative bidirectional promoters of 100–600 bp between predicted TSS (transcription start sites) of protein-coding genes in the Arabidopsis genome and further selected for those containing multiple (two or more) CACGTG motifs. This results in a list of 70 gene pairs (Table 1). The G-box ABRE is often found in combination with other motifs, coupling elements (CE) that can be based on or distinct from the G-box and also involved in the seed/ABA regulation. Therefore we searched PLACE [45] with all 70 intergenic regions focusing on DREs based on the CCGAC core and CE3s not derived from the ACGT core. Where these elements were also identified is indicated in Table 1.

**Table 1:** Putative bidirectional gene pairs identified with 100–600 bp between transcription start sites (TSS) and containing at least two G-Box elements

**FWgene**	**RVgene**	**len**	**DRE**	**CE3**	**FWgene annotation**	**RVgene annotation**	**FWlocation**	**RVlocation**
**AT1G04635**	AT1G04630	202	y		AtPOP5	maternal effect embryo arrest 4 (MEE4)	nucleolus	mitochondrion
**AT1G07510**	AT1G07500	347			FtsH10 protease	unknown protein	mitochondrion	cytosol
**AT1G07660**	AT1G07645	452			Histone superfamily	vicinal oxygen chelate (VOC) metalloenzyme	nucleus	cytosol
**AT1G07985**	AT1G07980	478			Expressed protein	nuclear factor Y, subunit C10	nucleus	plastid
**AT1G15330**	AT1G15320	333			Cystathionine beta-synthase (CBS)	unknown protein	cytosol	extracellular
**AT1G16740**	AT1G16730	378			Ribosomal protein L20	unknown protein 6 (UP6)	mitochondrion	nucleus
**AT1G19980**	AT1G19970	327			cytomatrix protein-related	ER lumen protein retaining receptor family	nucleus	ER
**AT1G28540**	AT1G28530	164			unknown protein	unknown protein	cytosol	plastid
**AT1G31420**	AT1G31410	259	y		LRR kinase (FEI1)	putrescine-binding transporter protein	membrane	plastid
**AT1G32560**	AT1G32550	367	y		LEA4-1	Ferredoxin C2	nucleus	plastid
**AT1G48840**	AT1G48830	264	y		unknown function (DUF639)	Ribosomal protein S7e family	membrane	cytosol
**AT1G50440**	AT1G50430	161			RING/FYVE/PHD zinc finger	DWARF 5 (DWF5) -STEROL DELTA7 REDUCTASE	nucleus	membrane
**AT1G52230**	AT1G52220	139			PHOTOSYSTEM I SUBUNIT H2	unknown protein	plastid	plastid
**AT1G54870**	AT1G54860	375			ChlADR aldehyde reductase	Glycoprotein membrane GPI-anchored	cytosol	extracellular
**AT1G56170**	AT1G56165	391			CCAAT motif binding complex	non-coding RNA	nucleus	cytosol
**AT1G61790**	AT1G61780	237			Oligosaccharyltransferase	postsynaptic protein-related	ER	nucleus
**AT1G65140**	AT1G65130	595			Ubiquitin c-terminal hydrolase	Ubiquitin c-terminal hydrolase	cytosol	nucleus
**AT1G71090**	AT1G71080	370			Auxin efflux carrier	RNA pol II transcription elongation factor	membrane	nucleus
**AT1G72030**	AT1G72020	434			Acyl-CoA N-acyltransferase	unknown protein	plastid	mitochondrion
**AT1G77370**	AT1G77360	307			Glutaredoxin	Tetratricopeptide repeat (TPR)-like	extracellular	mitochondrion
**AT2G20490**	AT2G20480	189			NOP10; EDA27 (RNA binding)	unknown protein	nucleolus	nucleus
**AT2G25890**	AT2G25880	470			Oleosin	Ser/Thr kinases - Ataurora2 (AUR2)	lipid body	nucleus
**AT2G29560**	AT2G29550	523			phosphoenolpyruvate enolase	beta-tubulin (TUB7)	cytosol	nucleus
**AT2G38040**	AT2G38025	385	y		acetyl-CoA carboxylase subunit	Cysteine proteinase superfamily	plastid	plastid
**AT2G38660**	AT2G38650	183			Amino acid dehydrogenase	galacturonosyltransferase 7 (GAUT7)	mitochondrion	golgi
**AT2G39460**	AT2G39450	331	y		60S ribosomal protein L23aA	manganese transporter	cytosol	golgi
**AT2G43190**	AT2G43180	187			AtPOP4 (RNA processing)	Phosphoenolpyruvate carboxylase family	nucleolus	plastid
**AT2G45740**	AT2G45730	556	y		peroxin11 (PEX11) family	eukaryotic initiation factor 3 ⊠ subunit	peroxisome	cytosol
**AT3G03160**	**AT3G03150**	**264**	**y**	**y**	**unknown protein**	**unknown protein**	**endomembrane**	**mitochondrion**
**AT3G03320**	AT3G03310	135			RNA-binding protein	lecithin:cholesterol acyltransferase 3	cytosol	plasma membrane
**AT3G12320**	AT3G12300	580	y		unknown protein	unknown protein	nucleus	cytosol
**AT3G13190**	AT3G13180	559			unknown function (DUF827)	rRNA small subunit methyltransferase B	nucleus	plastid
**AT3G14340**	AT3G14330	305			unknown protein	Tetratricopeptide repeat (TPR)-like	membrane	mitochondrion
**AT3G15290**	AT3G15280	304			3-hydroxyacyl-CoA DH	unknown protein	peroxisome	mitochondrion
**AT3G16010**	AT3G16000	197			PPR-like superfamily	plastid DNA-binding protein; MFP1	mitochondrion	plastid
**AT3G18215**	AT3G18210	245			unknown function, DUF599	2OG and Fe(II)-dependent oxygenase	membrane	nucleus
**AT3G26618**	AT3G26616	464			eukaryotic release factor 1-3	unknown protein	cytosol	cytosol
**AT3G52230**	AT3G52220	139			unknown protein	Kinase phosphorylation domain	plastid	nucleus
**AT3G53180**	AT3G53170	477			glutamate-ammonia ligases	Tetratricopeptide repeat (TPR)-like	cytosol	cytosol
**AT3G59500**	AT3G59490	190			HRF1 family protein	unknown protein	ER membrane	nucleus
**AT4G00030**	AT4G00026	168			Plastid-lipid associated (PAP)	SD3 (Segregation Distortion 3); TIM21	plastid	mitochondrion
**AT4G01270**	AT4G01265	577	y		RING/U-box superfamily	raffinose synthase family pseudogene	nucleus	NA
**AT4G02430**	AT4G02425	189	y		S/R-Rich Protein Splicing Factors	unknown protein	nucleus	nucleus
**AT4G11985**	AT4G11980	129			pre-tRNA/non-coding RNA	nudix hydrolase homolog 14	nucleus/cytosol	plastid
**AT4G16160**	AT4G16155	205	y		OEP16-S	dihydrolipoyl dehydrogenase	plastid	plastid
**AT4G17560**	AT4G17550	541	y		Ribosomal protein L19 family	glycerol-3-phosphate permease gene family	plastid	mitochondrion
**AT4G17730**	AT4G17720	440			syntaxin23	RNA-binding (RRM/RBD/RNP motifs) family	cytosol	cytosol
**AT4G18240**	AT4G18230	169			starch synthase 4 (SS4)	unknown protein	plastid	plasma membrane
**AT4G18370**	AT4G18360	447			DEG5 - photosystem II repair	Aldolase-type TIM barrel family	plastid	peroxisome
**AT4G19020**	AT4G19010	277			chromomethylase 2 (CMT2)	AMP-dependent synthetase and ligase family	nucleus	peroxisome
**AT4G21280**	AT4G21270	267			PsbQ subunit photosystem II	kinesin-like motor protein	plastid	nucleus
**AT4G23840**	AT4G23820	254			Leucine-rich repeat (LRR) family	Pectin lyase-like superfamily	cytosol	extracellular
**AT4G25140**	AT4G25130	366			Oleosin1	chloroplast methionine sulfoxide reductase	lipid body	plastid
**AT4G25580**	AT4G25570	561			cold acclimation protein (CAP160)	cytochrome b561	nucleus	plasma membrane
**AT4G31080**	AT4G31070	351			unknown function (DUF2296)	Tetratricopeptide repeat (TPR)-like	ER	cytosol
**AT4G33540**	AT4G33530	365			metallo-beta-lactamase family	K + UPTAKE PERMEASE 5 (KUP5)	plastid	plasma membrane
**AT5G05490**	AT5G05480	293			SYN1 (RAD21-like) gene	Peptide-N4-asparagine amidase A protein	nucleus	plasma membrane
**AT5G05987**	AT5G05980	509	y		prenylated RAB acceptor 1.A2	folylpolyglutamate synthetase isoform	membrane	mitochondrion
**AT5G07320**	AT5G07315	363			Mito ATP-Mg/Pi transporter	pre-tRNA/non-coding RNA	mitochondrion	nucleus/cytosol
**AT5G10080**	AT5G10070	409			Eukaryotic aspartyl protease	RNase L inhibitor protein-related	membrane	cytosol
**AT5G10745**	AT5G10740	275			unknown protein	Protein phosphatase 2C family protein	membrane	nucleus
**AT5G16760**	AT5G16750	331			inositol-trisphosphate 5/6-kinase	TORMOZEMBRYO DEFECTIVE (TOZ)	cytosol	nucleolus
**AT5G37350**	AT5G37340	232			RIO1 kinase	ZPR1 zinc-finger domain protein	nucleus	cytosol
**AT5G51540**	AT5G51530	529			Zincin-like metalloproteases	Ubiquitin c-terminal hydrolase	plastid	cytosol
**AT5G54062**	AT5G54060	424			unknown protein	anthocyanin 3-O-glucoside	extracellular	membrane
**AT5G54970**	AT5G54960	449			unknown protein	pyruvate decarboxylase-2	cytosol	cytosol
**AT5G61940**	AT5G61930	481	y		Ubiquitin c-terminal hydrolase	ACCUMULATION OF PHOTOSYSTEM ONE 3	nucleus	mitochondrion
**AT5G62490**	AT5G62480	537			AtHVA22 family	glutathione transferase	extracellular	cytosol
**AT5G64220**	AT5G64210	582			Calmodulin-binding activator	isoform of alternative oxidase	nucleus	mitochondrion
**AT5G67230**	AT5G67220	514			GT43 glycosyltransferase family	FMN-linked oxidoreductase superfamily	mitochondrion	mitochondrion

Presence of a DRE and/or CE3 motif (not derived from an ACGT-core sequence) also indicated by ‘y’.

The only gene pair with a DRE and a CE3 element is highlighted.

The cellular localisation of each gene product was noted based on predictions using the TargetP tool and individual gene profiles available on TAIR. Proteins are predicted to lie in all main cell compartments and divergent gene pairs could be in the same or different locations. However, 25% were predicted to be localized to the plastid, 20% to the endomembrane/secretory system, 14% to the mitochondria and the remaining 40% to other components including cytoplasm and nucleus. At the functional or activity level, 33% encode enzymes. Previous studies have noted an enrichment for enzymes/metabolism and organellar localisation in the human genome [53,54] and the seed development process is naturally accompanied by extensive metabolic fluctuation [33,55,56]. 24% of genes are potentially involved in direct gene expression regulation, DNA/RNA binding and processing. Some of the genes identified have already been shown to be involved in embryo/endosperm development (At1g04630 and At2g20490 were identified as *MEE4* and *EDA27*, respectively, by Pagnussat *et al.*[57] and other genes are more obviously associated with some form of light-regulation (At4g21280, *PsbQ* subunit) [58]. Interestingly, *MEE4* shares its bidirectional promoter with AtPOP5 which was shown to physically interact with AtPP30, involved in female gametophyte development, in an RNase P/MRP complex [59]. The *TORMOZ* and *AURORA* genes (At5g16750 and At2g25880) are involved in embryo development [60,61]; *SYN1* (At5g05490) is essential for meiosis [62]; SD3 and DWF5 are membrane proteins involved in seedling development [63,64]. Other genes have been shown experimentally to be responsive to drought or ABA such as LEA4-1 and OEP16-S (At1g32560 and At4g16160; [33,65]; Additional file 1: Figure S2B and Additional file 1: Figure S1A). The gene pairs were also examined for the extent of co-expression using AtGenExpress Visualisation Tool [38-40] which showed that while in some cases the genes showed very similar patterns of expression, there were also cases where the expression patterns of both genes differed significantly in terms of both temporal and spatial patterns and also in terms of their response to various stresses (Additional file 1: Figure S2). Several parameters can be taken into account when assessing if a gene pairs’ products might be directly linked functionally such as the extent of co-expression and the subcellular co-localization of the gene pair products. Only three gene pairs have products that are predicted to be targeted to the same organelle (not counting cytosolic predictions). At1g52230 and At1g52220, encoding the PSI subunit H and an unknown protein respectively, have identical expression patterns according to AtGenExpress (Additional file 1: Figure S2A) and are localized in the thylakoid system of the plastid. Furthermore we found that a previous analyses of a chloroplast protein interaction network [66] had predicted an interaction between these proteins and that the localization of the unknown At1g52220 product to the thylakoid was confirmed as well as a physical interaction with the D subunit of PSI.

CE3 coupling elements are rare in the Arabidopsis genome [26,67] and while CE3-like elements have been described [47,68], this is the first report of a consensus CE3 element in Arabidopsis. Therefore we chose to focus on the only pair of genes containing both a DRE and a CE3 element (Highlighted in Table 1). In addition, this pair At3g03150-At3g03160 has SALK T-DNA insertion lines available within the coding regions to enable preliminary functional analyses of the genes.

### At3g03150 and At3g03160 are transcribed from a putative bidirectional promoter and encode novel plant-specific proteins

**Figure 1:**
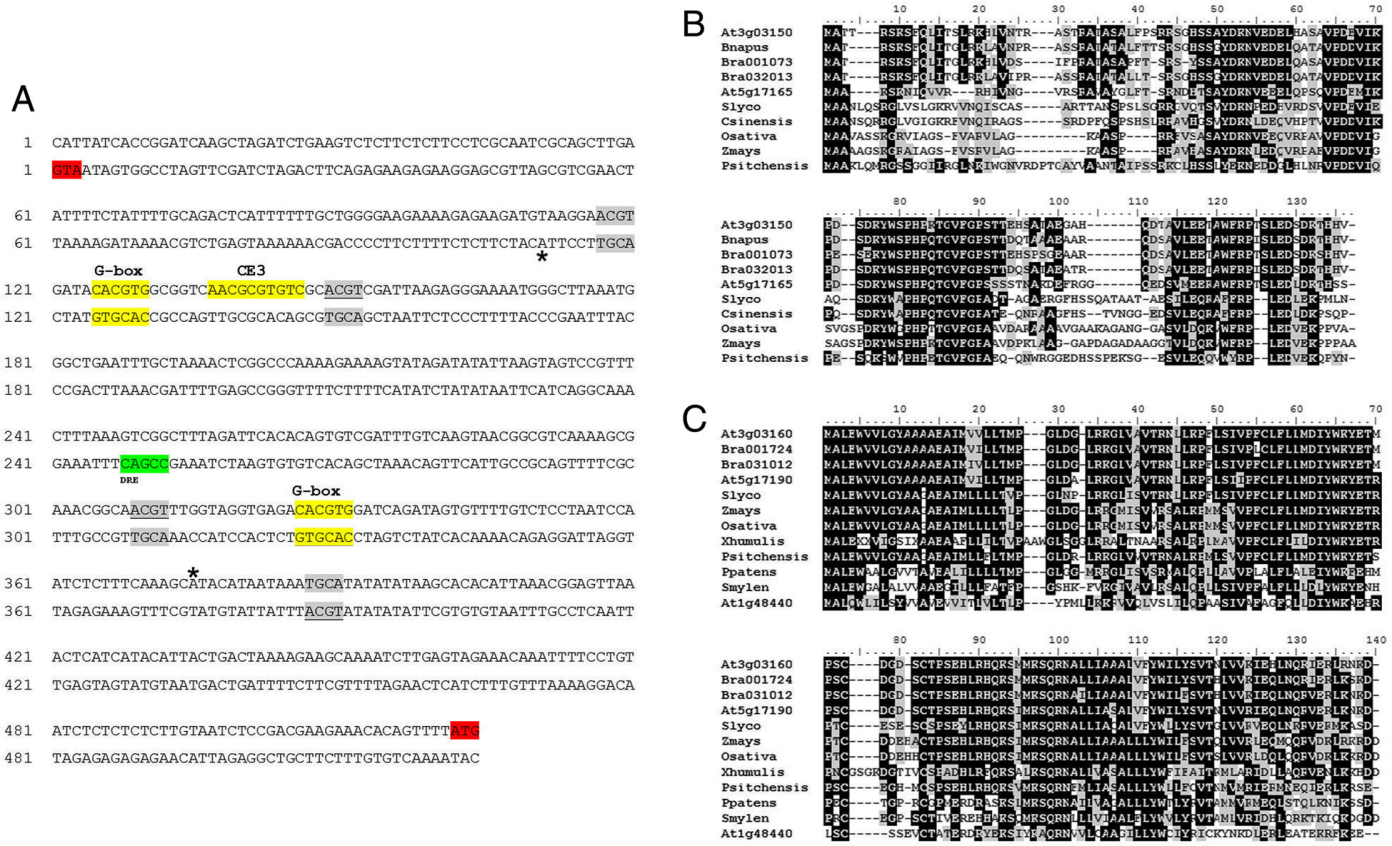
**Features of the At3g03150-At3g03160 gene pair. (A)** Intergenic promoter region showing G-box hexamers (yellow), the CE3 (yellow) and DRE (green) elements. TSS sites are indicated by asterisks. Other ACGT motifs are in grey. **(B)** At3g03150 amino acid alignment with orthologues across the angiosperms. **(C)** At3g03160 amino acid alignment with orthologues including bryophytes and lycophytes. Accession numbers of the sequences used for the alignments are shown in Additional file 2: Table S1.

Analysis of the promoter region revealed that divergent ORFs (At3g03150 and At3g03160) are separated by 518 bp, suggesting that both genes possibly share the same promoter and that expression may be co-regulated. The promoter contains several ACGT elements and a 100% match to the CE3 ABA responsive element in the *HVA1* promoter of barley [69]; (Figure 1A).

The protein sequence of At3g03150 had no familiar domains or motifs that would indicate a function. Localization prediction using PSORT and TargetP programs indicated (with 75% and 91% probability, respectively) mitochondrial localization. The predicted cleavage site matches consensus sites and the targeting region is rich in serine. Overall the 120 amino acid protein is composed of almost 15% serine and threonine residues. Over 37% are PEST residues of which 16% are serine alone. A high PEST content is indicative of proteins with high turnover and indicates that the protein may be unstable. The gene’s structure is particularly striking showing the presence of one intron of over 1.2 kb and with the first exon discretely encoding the target sequence. To test the validity of the localization prediction of At3g03150, a translational protein fusion with GFP at the C-terminal of the protein was constructed to test for mitochondrial localization. Results in Additional file 1: Figure S3A confirm localization of the protein to the mitochondrion.

The divergent gene, At3g03160, is also of unknown function but contains three transmembrane domains (TMHMM Server v2.; [46]; Additional file 1: Figure S3B) and is homologous to a dehydration-induced transcript from the resurrection plant *Xerophyta humilis*[70] (Figure 1C). The intergenic promoter contains a consensus DRE (Figure 1A). TargetP predicts a signal peptide and so it is likely the protein is part of the secretory pathway.

In addition, there are orthologues of At3g03150 in vascular plants only while At3g03160 has orthologues in *Selaginella* and *Physcomitrella patens* (Figure 1B,C). The strong preservation of certain blocks of sequence between At3g03150 and orthologues identified in Genbank, even before the emergence of the flowering plants (represented by the pine sequence), suggests that these putative active sites are well-conserved across a broad evolutionary time-frame (Figure 1B). The level of amino acid identity between At3g03160 orthologues across the embryophyta (land plants) is striking (Figure 1C).

Both genes have paralogues on chromosome 5 and appear to be the result of a genomic duplication previously identified [71,72]. The corresponding region on chromosome 5 preserves the divergent arrangement of the genes but with two other genes, At5g17170 and At5g17180, intervening (Additional file 1: Figure S3C). These intervening genes are single-copy. At3g03160 is homologous to At5g17190. In the case of At5g17165, the modular nature of the gene structure with the first exon encoding the targeting peptide is maintained.

BLAST analysis against the rice and Brassica genomes revealed that the bidirectional arrangement of these two genes is only conserved in *Brassica rapa*. The duplication corresponding to At3g03160/At5g17190 and At3g03150/At5g17165 occurred before *Brassica/Arabidopsis* split and a further duplication in *Brassica* produced two At3g03150 and two At3g03160 paralogues (Figure 1B,C).

### Both genes share a similar general expression pattern but are not identical

**Figure 2:**
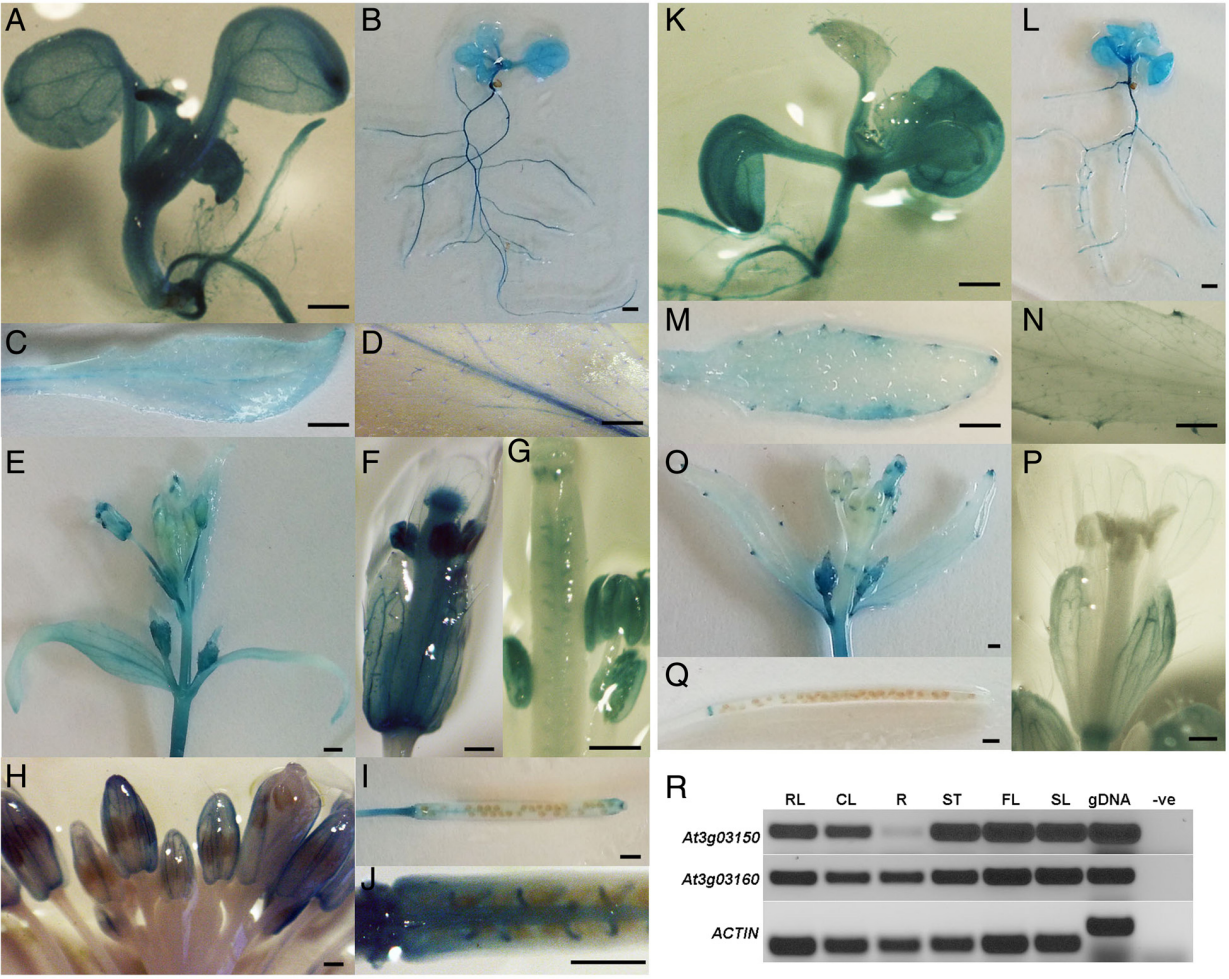
**Expression patterns of At3g03150 and At3g03160.** Patterns of GUS distribution in vegetative and reproductive tissues of plants transformed with Promoter-GUS fusions for At3g03150 **(A-J)** and At3g03160 **(K-Q). (R)** RT-PCR survey on various Arabidopsis tissues for the detection of At3g03150 and At3g03160 transcripts. Actin was used as control. RL, rosettleaf; CL, cauline leaf, R, root of mature plant; ST, stem; FL, flower; SL, silique; gDNA, genomic DNA; -ve, negative control (water). Scale bars 1 mm **(A, B, E, I, K, L, O, Q)**; 0.5 cm **(C, D, M, N)**; 0.5 mm **(F, G, H, P)**; 0.2 mm **(J)**.

A general RT-PCR survey suggested that both genes were transcribed in similar spatial patterns (Figure 2R) and Yang *et al.*[73] had previously listed these genes as being co-expressed divergent genes. However, data from AtGenExpress revealed more subtle differences between the At3g03150-At3g03160 gene pair. Therefore, the intergenic promoter was used to make transcription-fusions with the GUS reporter gene in both orientations and the expression pattern was monitored in detail (Figure 2). At the seedling stage, GUS expression in both orientations was high and ubiquitous though in older seedlings the expression of At3g03160 appeared to be more localized to the tips of the main and lateral roots and in the initiating lateral buds. In mature leaves the expression of At3g03150 was obvious in the vasculature while At3g03160 expression was very noticeable in the hydathodes. Both genes were expressed extensively in floral buds, open flower and fruit tissues but there was significant variation through development. Both genes were expressed in outer whorls early in development but At3g03160 became highly localized to the abscission zones and pedicel as the flower matured. Furthermore, expression in stigmatic tissues as well as in the anthers and pollen was much stronger in the At3g03150 orientation. There was also strikingly strong GUS expression observed in the funiculus in the At3g03150 orientation (Figure 2).

The expression patterns of the paralogues At5g17165 and At5g17190, respectively, were also checked by RT-PCR with gene-specific primers. At5g17165 produced a transcript spanning the two exons which was detectable at a level significantly less than that of At3g03150 except in flower tissues (Additional file 1: Figure S3D). At5g17190, like At3g03160 is also highly expressed in roots and developing seeds (Additional file 1: Figure S3D).

### The genes respond differently to stresses

**Figure 3:**
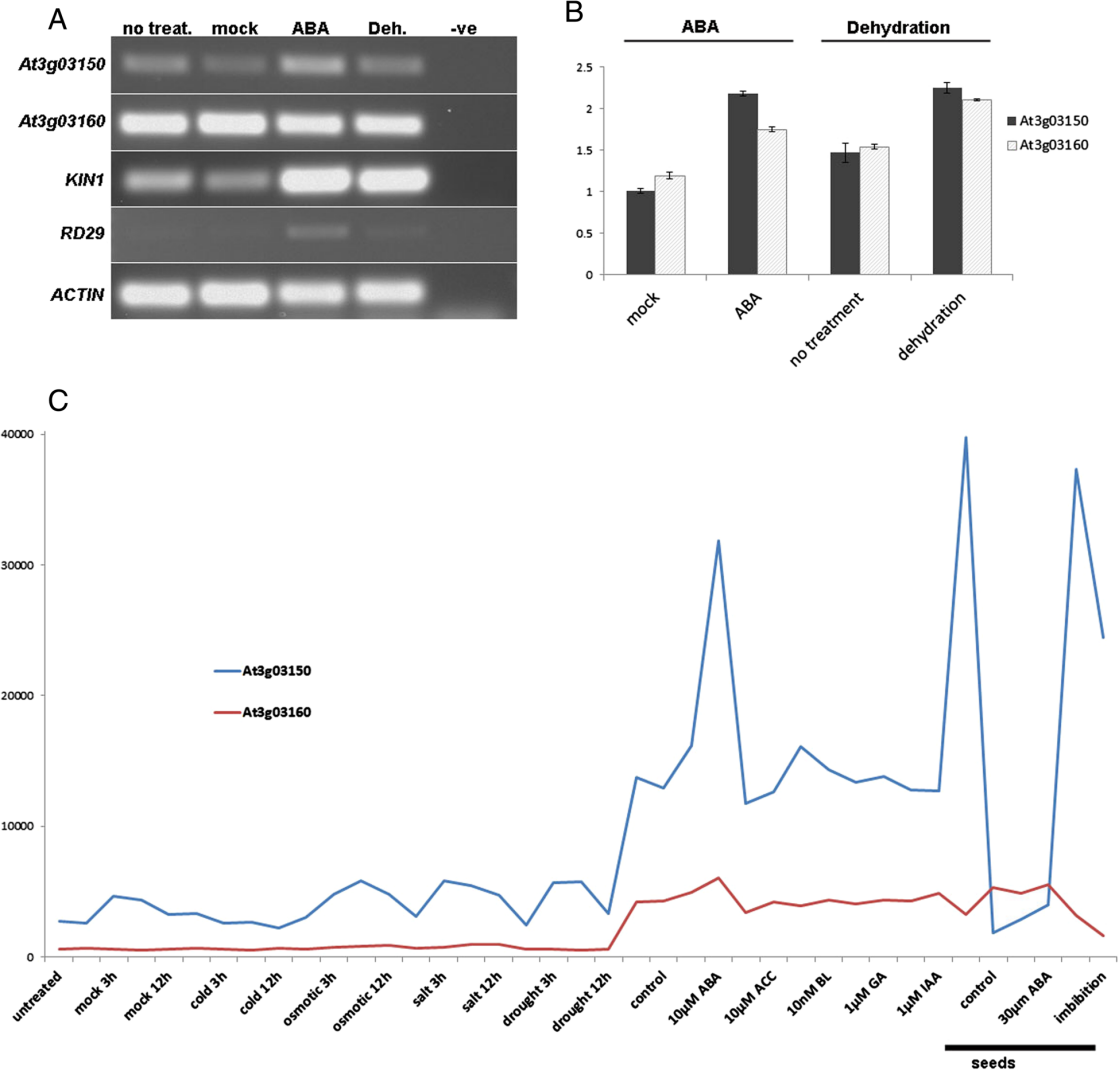
**Response of At3g03150 and At3g03160 to various stresses. (A)** RT-PCR of At3g03150 and At3g03160 after treatment of 3-week old seedlings with exogenous ABA (ABA) and dehydration (Deh.) *KIN1* and *RD29* are used as positive controls. **(B)** qRT-PCR of At3g03150 and At3g03160 in seedlings treated with exogenous ABA and dehydration normalized to 18S gene expression. **(C)** AtGenExpress expression profiles for both genes under stress and hormone treatments in seedlings except where indicated otherwise.

The presence of consensus and adjacent ABRE and CE3 elements in the intergenic region strongly suggested that At3g03150 would be regulated by ABA. In addition, the AtGenExpress profile for At3g03150 also indicated that the gene is up-regulated in ABA experiments. To confirm this experimentally, expression of At3g03150 was examined by RT-PCR in 3-week old seedlings subjected to addition of exogenous ABA. Genes known to respond to ABA, *KIN1* and *RD29* were used as positive controls and Figure 3A shows that At3g03150 responded to ABA treatment. In contrast we did not detect any visible response of At3g03160 to ABA treatment nor any response to dehydration treatment using standard RT-PCR. We had expected to detect some response of At3g03160 to dehydration based on its homology to a drought induced transcript [70] and the presence of a putative DRE element in the promoter (Figure 1A). We therefore repeated the analyses using qRT-PCR (Figure 3B) on both genes normalized to the 18S reference gene. This showed the ABA response already seen in At3g03150 but also indicated a weaker response from At3g03160. In addition, both genes were upregulated under dehydration.

Data pertaining to stress and hormone treatments was also extracted from AtGenExpress (Figure 3C). This showed obvious ABA responsiveness in At3g03150 but also a weaker response of At3g03160 at 10 μM ABA. At3g03150 responded to drought treatments at 3 and 6 hours but no discernible response was seen for At3g03160 – however there is an obvious reduction in At3g03160 expression on seed imbibition suggesting that the gene may respond negatively to hydration in this context.

### Both genes play a role in seed development

**Figure 4:**
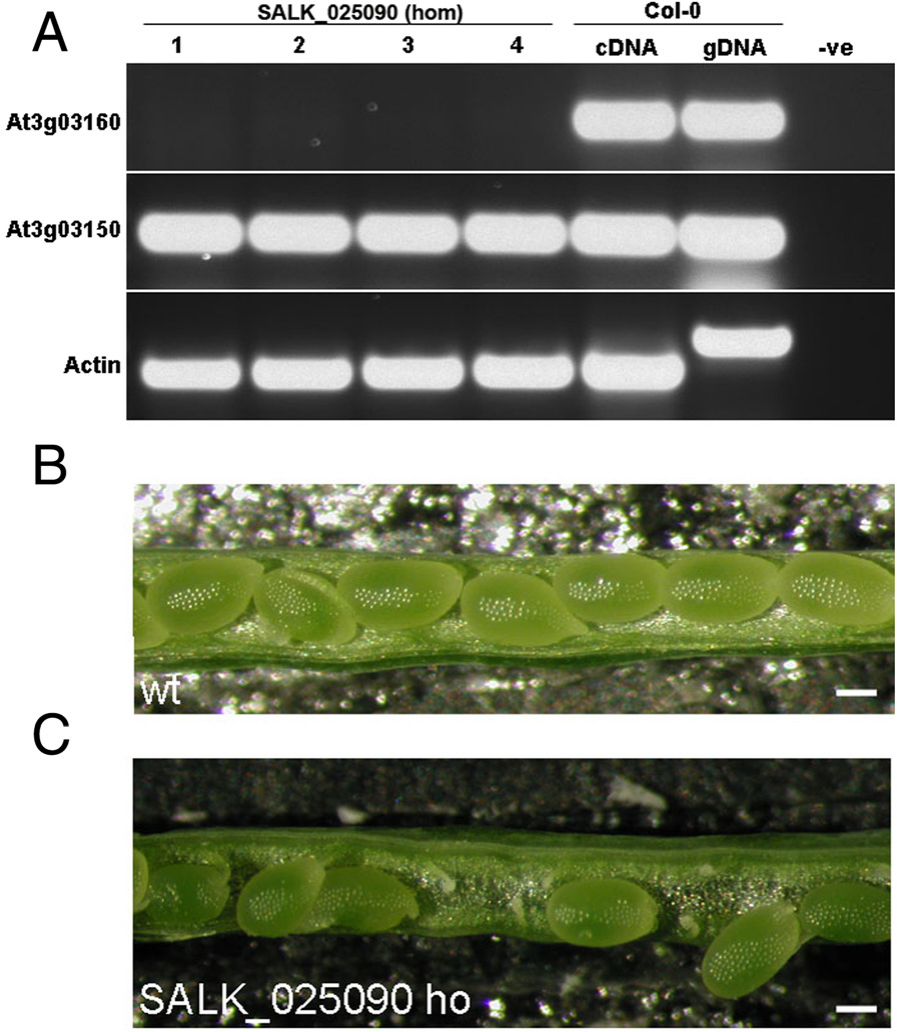
**Characterization of the At3g03160 T-DNA line SALK_025090. (A)** RT-PCR showing no expression of At3g03160 in the homozygous SALK_025090 line but high expression of At3g03150. 1–4, cDNA from individual homozygous plants. **(B)** Wild-type and **(C)** SALK_025090 siliques showing lower seed set. Scale bars 50 μm.

A homozygous SALK T-DNA insertion line (SALK_025090) was obtained for At3g03160 and analyzed. Genotyping and sequencing confirmed that the T-DNA insertion was located 119 bp downstream of the start codon within the coding sequence and RT-PCR showed that there was no expression of the At3g03160 gene in this insertion line (Figure 4A). Expression of the adjacent divergent At5g03150 was also tested to make sure that the expression levels of this gene were not affected in the At3g03160 insertion line (Figure 4A). Phenotypic analysis revealed a significantly lower seed set in the siliques (Table 2, Figure 4B). Specifically, there was an increase in both the number of unfertilized ovules and aborted seeds.

**Table 2:** Seed counts from siliques of wild-type Col-0 and T-DNA insertion lines for At3g03150 and At3g03160

**Line**	**% Unfertilised ovules**	**% Early aborted seeds**	**% Late aborted seeds**	**% Normal dev. seeds**
**Col-0**	3.22	0.63	0.58	95.56
**SALK_121507(het)**	58.21	0.63	6.97	34.19
**SALK_025090(hom)**	22.15	0.74	5.29	71.18

**Figure 5:**
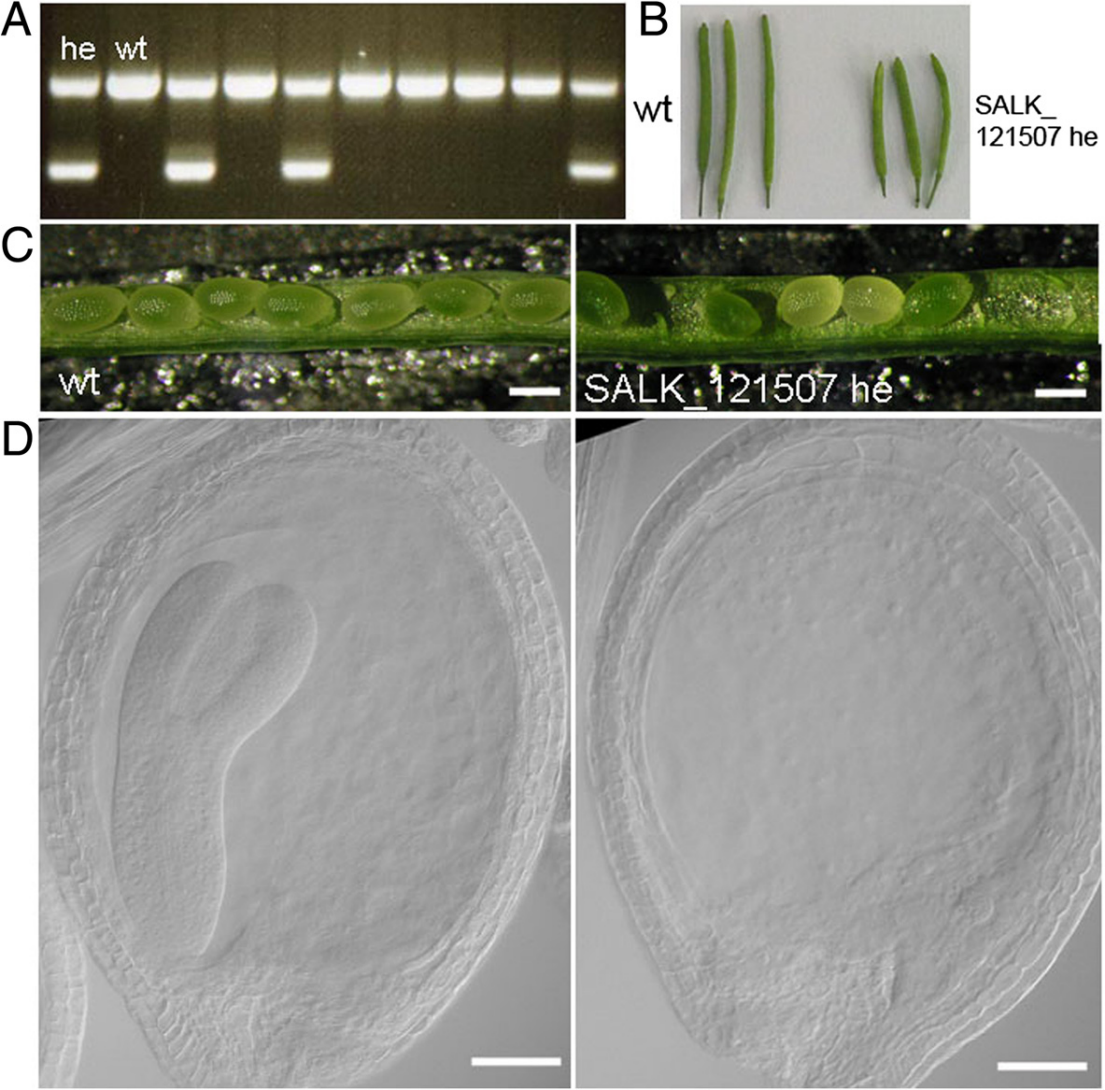
**Characterization of the At3g03150 T-DNA line SALK_121507. (A)** Genotyping of SALK-121507 showing identification of heterozygous (double bands) and wild-type plants (single bands). **(B)** Shorter siliques in heterozygous SALK_121507 plants compared to wild-type. **(C)** Unfertilisated ovules and late aborted seeds (white) in SALK_121507 compared to wild-type. **(D)** Cleared adjacent wild-type (left) and aborted seeds (right) in a SALK_121507 silique. Scale bars 100 μm **(C)**; 50 μm **(D)**.

**Figure 6:**
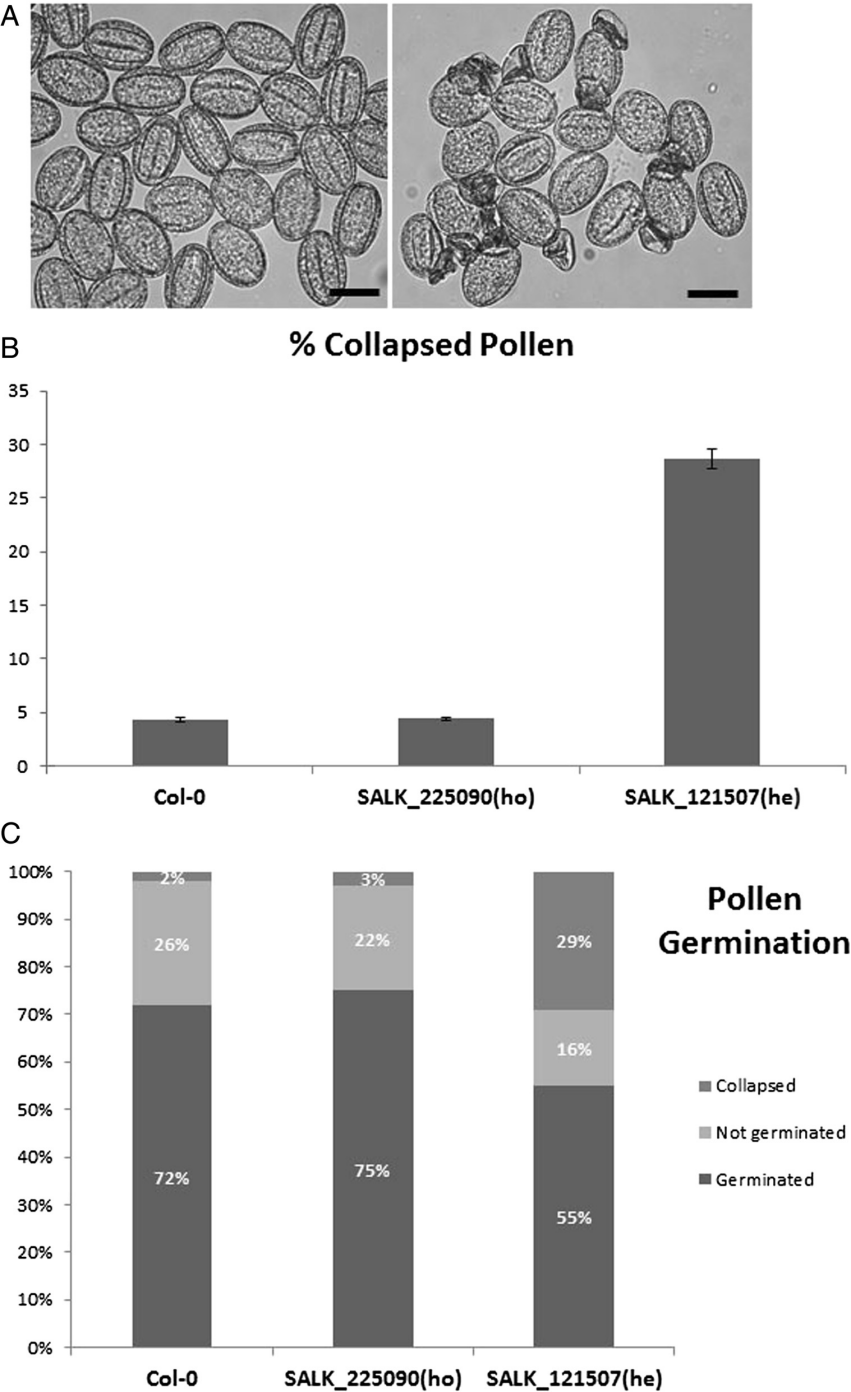
**Analyses of pollen in SALK_121507 heterozygote and SALK_025090 homozygote lines. (A)** Comparison of wild-type (left) and SALK_121507 heterozygote pollen (right) showing a high percentage of collapsed pollen grains. Scale bars 20 μm. **(B)** Percentage of collapsed pollen in wild-type, SALK_025090 homozygous and SALK_121507 heterozygous lines. Error bars represent ± SE. **(C)** Pollen germination assay highlighting the lower percentage of germinating pollen grains in heterozygous SALK_121507 lines compared to the wild-type and homozygous SALK_025090.

SALK T-DNA lines were also obtained and analyzed for At3g03150. SALK_121507 was genotyped and sequenced and it was confirmed that the insertion was 3 bp downstream of the ATG codon – the only T-DNA line with an insert in the coding region of the gene (Figure 5A). This line could not be propagated as a homozygous line but the heterozygous lines segregated with a silique and ovule phenotype (Figure 5B-D). Siliques of the heterozygotes were shorter than wild-type. 58.21% of ovules appeared to be unfertilised (compared to 3.22% for the wild-type). A higher than normal percentage of late aborted seeds were also observed compared to the wild-type (6.97%) (Table 2). Figure 5C shows the presence of large white ovules in the siliques of heterozygous plants adjacent to normal green ovules. Microscopic analysis of cleared samples showed that these ovules cease developing even before true globular stage (Figure 5D) when the adjacent ovules have developed to walking stick stage. There also appears to be a defect in endosperm development at the chalazal end. Maternal tissues appear to develop normally and the aberrant embryo and endosperm does not affect ovule growth and size. The presence of unfertilized ovules in both insertion lines prompted an analysis of pollen to determine if that might be contributing to the failure of fertilization. Pollen from the SALK_025090 homozygotes and the SALK_121507 heterozygotes stained with DAPI to assess grain morphology and FDA to check pollen grain viability, while pollen germination assays were also performed. The results suggest that the SALK_025090 pollen is normal when compared to wild-type but that the pollen of the SALK_121507 line is defective with a relatively high percentage (30%) of collapsed and non-germinating pollen grains produced (Figure 6). This correlates with expression patterns of the genes as GUS expression was detected in pollen for the At3g03150 promoter only (Figure 2F,G).

Selfing of the SALK_121507 heterozygotes produced a consistent ratio of wild-type to heterozygote of 1:0.7 in progeny populations suggesting that it was a gametophytic mutant. This was tested further by crossing the heterozygote to wild-type plants as both pollen donor and recipient. Screening of the F1 population of the reciprocal out-crosses to the wild-type for the presence of the T-DNA insertion revealed that there is a slight reduction in the transmission efficiency both through the male and the female (TE_male_ = 70.5%, TE_female_ = 88%, Additional file 4: Table S3). The fact that, despite the significant transmission through both gametophytes, homozygous plants for the T-DNA insertion were never recovered after selfing indicates that this mutation causes zygotic lethality.

## Discussion

Though recognized as being a common phenomenon in animal and plant genomes based on bioinformatics analyses, there is currently no example in the literature that uses a targeted analysis of bidirectional promoters with associated functional characterization of the genes involved. Here we have conducted a targeted search for putative bidirectional promoters involved in seed development using limited examples from the literature and characterized one of the promoters and associated gene pairs in detail, including preliminary functional analyses. This approach could complement conventional screening approaches in the search for genes involved in seed-associated processes [57,74-77] and/or for genes regulated by hormones and stresses [26] and our dataset contains some genes also identified in these screens [26,57]. The bidirectional promoter structure suggests co-ordination of expression and indeed much of the bioinformatics analysis to date includes evidence of co-expression of diverging genes based on the large volume of transcriptomic data available. AtGenExpress analysis of the 70 bimotif flanking genes shows some that mirror each other and others that are very different (Additional file 1: Figure S1; Additional file 5: File S1). Bioinformatics analyses have tended to focus on the co-expression or even common GO categorization. With the large datasets this is understandable but if focusing on specific subsets it might be possible to tease apart other cases where protein function or expression pattern is not obviously similar. Co-expression does not necessarily mean spatial and temporal expression similarities but could also involve a coordinated response to stress that could be tissue-distinct, and indeed even organelle -distinct. These coordinated responses may be mediated through promoter *cis*-elements. It has been found that by integrating known *cis*-elements with co-expression increases the reliability of associated gene function prediction [78]. Though 40% of the genes in Table 1 are predicted to be mitochondrial or plastid-localised, there are only three cases where both genes are predicted to be localized to the same organelle. In the case of the oleosin and PMSR genes, the AtGenExpress expression profiles diverge considerably with oleosin being seed-specific and PMSR expressed ubiquitously (Additional file 1: Figure S1B). In addition, oleosin is ABA-induced – often characteristic of a gene highly expressed in maturing seeds – while PMSR responds to oxidative stress, a natural consequence of seed maturation [36,56]. While the At3g03150-At3g03160 intergenic region contains two ABRE palindromic CACGTG hexamers, the adjacent nucleotides vary in either orientation (which has shown to be an important determinant of binding specificity; [24,25]) and the CE3 element is unidirectional. As might be expected therefore this variation is reflected in expression patterns and function, though both genes appear to affect aspects of seed development.

The bidirectional arrangement might be a particularly efficient way to mediate concerted or complementary response to stresses or environmental stimuli such as light or hormones. Bondino and Valle [12] pointed out that plants being sessile may need sophisticated means of coordinating gene expression responses to various stresses. In addition to the promoters coordinating responses to varied stresses and developmental signals, the localization of the gene products to varied organelles reflect a means of coordinating the intracellular interactions. Stresses such as drought, cold and salinity have shared and distinct signaling pathways, some of which are ABA-dependent [18]. In the case of At3g03150-At3g03160, the former is strongly regulated by ABA and located in the mitochondrion while the latter has a weaker response to ABA and is membrane-bound (though may be regulated by drought based on the presence of a DRE element and homology to a *X. humilis* desiccation-induced transcript) [70]. In the case of At1g07645 in Table 1 it is also homologous to a desiccation-induced *X. humilis* metalloenzyme but its expression was not affected in Arabidopsis seedlings under drought conditions [79]. Zhang *et al.*[26] searched for ABRE *cis*-elements in promoters to identify genes involved in associated stress responses but did not include any selection for potential bidirectionality in the promoters. Despite this, within the list of the top 40 predicted ABA/stress responsive genes in this study, 4 pairs of genes and another gene (23%) are also on our list in Table 1 and a further two pairs of divergent genes are also included (these were not on our list because the distance was slightly larger between genes and the ABRE search was not restricted to the CACGTC motif).

Co-regulation or co-ordination of the expression of multiple genes has been described in other arrangements and contexts. Operon structures, once thought exclusive to prokaryotes, have been found in biosynthetic pathways in plants (summarized by DellaPenna and O’ Conner [80]) coordinating the expression of genes with distinct functions but in a common biosynthetic pathway. Bidirectional promoters may constitute another means of coordinated expression in eukaryotes [53].

Analyses of the gene pairs spanning a putative bidirectional promoter may also help uncover functions for the vast array of unknown genes that remain to be characterised [53]. An initially “unknown” gene identified with a TSS 200 bp upstream and divergent to the *PARKIN* gene (a ubiquitin E3 ligase determining aspects of parkinsonism), *PACRG* (PArkin Co-Regulated Gene; [81]), was shown to share a common molecular pathway [82]. We were intrigued to find that in the case of the At1g52230 and At1g52220 gene pair, encoding the PSI subunit H and an unknown protein respectively, identified here (Table 1), a previous construction of a chloroplast protein interaction network had predicted an interaction between these proteins and a physical interaction of At1g52220 with a PSI subunit was confirmed [66].

There are still thousands of genes for which there is no definitive function assigned and this can only be done by careful experimental examination of individual genes in the laboratory at multiple levels – gene sequence, expression and function. In the *Arabidopsis* genome at least 40% of genes still have no determined function [83] and 20% of the mitochondrial proteome consisted of unknown proteins, many plant-specific [84]. Analyses of previously uncharacterised and plant-specific genes such as mitochondrial At3g13150 and transmembrane At3g03160 help accelerate these potential discoveries. Though we do not know what the activities of the encoded proteins are, we have described preliminary evidence of a common involvement in aspects of seed development.

## Conclusions

Bidirectional promoters are common in genome sequence but understudied experimentally, particularly in plants. Focusing on the G-box promoter motif, CACGTG, we performed a targeted identification of a subset of putative bidirectional promoters to identify genes involved in seed development and to investigate possible coordinated responses of gene pairs to conditions important in seed maturation such as desiccation and ABA-regulation. We further characterized a pair of genes sharing an intergenic region that also contained a CE3 element and describe preliminary functional data implicating two small, previously uncharacterized, plant-specific proteins in Arabidopsis seed development and stress responses.

## Competing interests

The authors declare that they have no competing interests.

## Authors’ contributions

SK carried out gene expression and T-DNA line characterization; KL performed the bioinformatics work identifying the intergenic regions; RH initiated the T-DNA line and gene sequence analyses; PR performed protein localization experiments; TP contributed to RT-PCR gene expression and T-DNA line analyses; SK and SD designed the study and drafted the manuscript. All authors read and approved the final manuscript.

## Supplementary Material

Additional file 1 **Figure S1.** Intergenic promoter regions and AtGenExpress profiles of selected gene pairs. **(A)** At4g16155-At4g16160 and **(B)** At4g25130-At4g25140. G-box hexamers (yellow) and storage protein (green) elements are highlighted in the intergenic promoter region, while other ACGT motifs are shown in grey. Development and hormone datasets from AtGenExpress were plotted with the vertical axis showing expression levels in a logarithmic scale. **Figure S2.** AtGenExpress profiles for At1g52220 -At1g52230 and At1g32550-At1g32560. **(A)** At1g52220 -At1g52230 profiles showing identical expression patterns and stress responses. **(B)** At1g32550-At1g32560 profiles with significantly different expression patterns and responses to various stresses. The values that were used were extracted from developmental, abiotic stress, hormones and light datasets with vertical axis showing expression levels in logarithmic scale. **Figure S3. (A)** Localisation of At3g03150 to the mitochondrion. Scale bar 20 μm. **(B)** Predicted transmembrane regions for At3g03160 using the TMHMM Server 2.0. **(C)** Schematic showing the organization of At3g03150 and At3g03160 paralogues on chromosome 5 (At5g17165 and At5g17190, respectively). **(D)** RT-PCR survey for the expression of the paralogues At5g17165 and At5g17190 using the same tissues as in Figure 2. RL, rosette leaf; CL, cauline leaf; R, root; ST, stem; FL, flower; SL, silique; gDNA, genomic DNA; -ve, negative control (water). Click here for file

Additional file 2: Table S1 Accession numbers of sequences used in Figure 1B-C. Click here for file

Additional file 3: Table S2 Sequences of all primers used in the present study. Click here for file

Additional file 4: Table S3 Transmission efficiency through the male and female gametophyte in SALK_121507. Click here for file

Additional file 5: File S1 AtGenExpress data extracted via the AVT. The data were used to make graphs in Additional file 1: Figures S1 and S2 and Figure 3. Click here for file
