# Supplementary figures and images for: Bidirectional promoters in seed development and related hormone/stress responses
Source: BMC Plant Biol. 2013 Nov 22;13:187. doi: 10.1186/1471-2229-13-187 (PMC4222868; doi:10.1186/1471-2229-13-187)

**Figure S1**


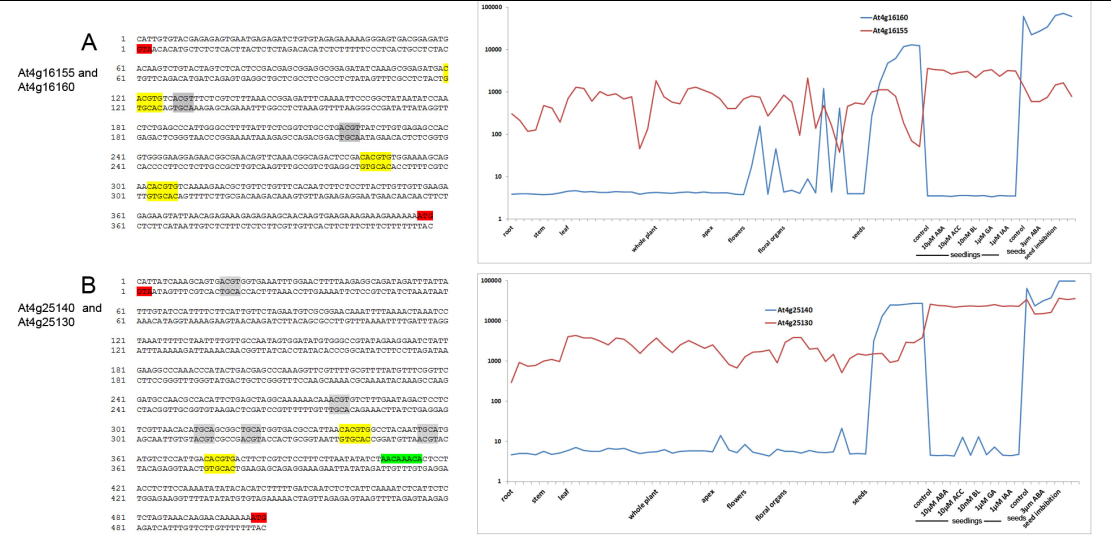


**Figure S2**


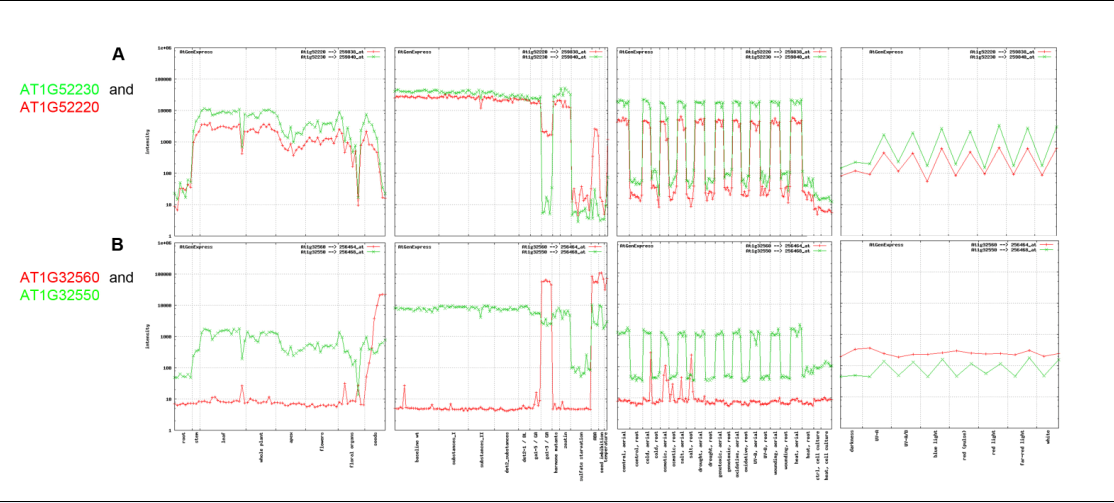


**Figure S3**

**
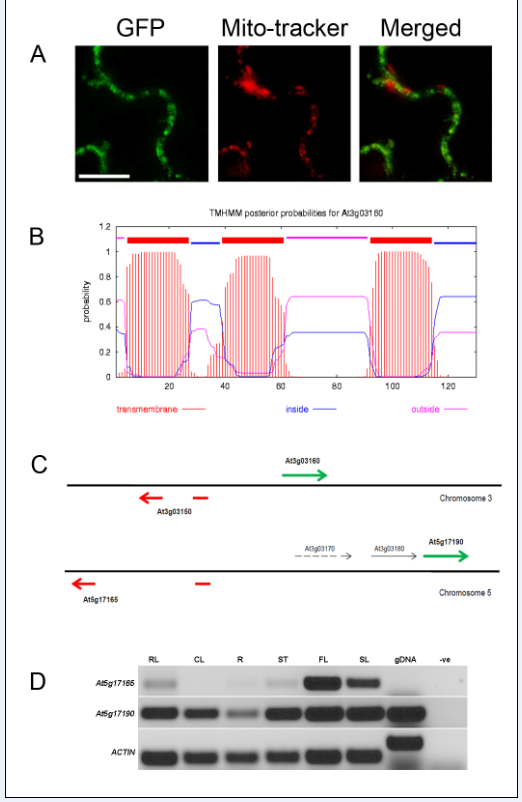
**

Supplement: Additional file 1 — Figure S1. Intergenic promoter regions and AtGenExpress profiles of selected gene pairs. (A) At4g16155-At4g16160 and (B) At4g25130-At4g25140. G-box hexamers (yellow) and storage protein (green) elements are highlighted in the intergenic promoter region, while other ACGT motifs are shown in grey. Development and hormone datasets from AtGenExpress were plotted with the vertical axis showing expression levels in a logarithmic scale. Figure S2. AtGenExpress profiles for At1g52220 -At1g52230 and At1g32550-At1g32560. (A) At1g52220 -At1g52230 profiles showing identical expression patterns and stress responses. (B) At1g32550-At1g32560 profiles with significantly different expression patterns and responses to various stresses. The values that were used were extracted from developmental, abiotic stress, hormones and light datasets with vertical axis showing expression levels in logarithmic scale. Figure S3. (A) Localisation of At3g03150 to the mitochondrion. Scale bar 20 μm. (B) Predicted transmembrane regions for At3g03160 using the TMHMM Server 2.0. (C) Schematic showing the organization of At3g03150 and At3g03160 paralogues on chromosome 5 (At5g17165 and At5g17190, respectively). (D) RT-PCR survey for the expression of the paralogues At5g17165 and At5g17190 using the same tissues as in Figure 2. RL, rosette leaf; CL, cauline leaf; R, root; ST, stem; FL, flower; SL, silique; gDNA, genomic DNA; -ve, negative control (water). [file 1471-2229-13-187-S1.doc]
