# Supplementary material for: Bidirectional promoters in seed development and related hormone/stress responses
Source: BMC Plant Biol. 2013 Nov 22;13:187. doi: 10.1186/1471-2229-13-187 (PMC4222868; doi:10.1186/1471-2229-13-187)
Supplement: Additional file 2: Table S1 — Accession numbers of sequences used in Figure 1B-C. [file 1471-2229-13-187-S2.docx]

**Additional file 2: Table S1. Accession numbers of sequences used in the amino acid alignments of Figure 1b-c.**

| Species | At3g03150 | At3g03160 |
| --- | --- | --- |
| *Brassica napus* | AAP37967 |  |
| *Zea mays* | ACF84353 | ACG24668 |
| *Solanum lycopersicon* | XP_004235359 | XP_004250171 |
| *Oryza sativa* | NP_001053740 | NP_001150948 |
| *Camellia sinensis* | AET97665 |  |
| *Xerophyta humulis* |  | AAT45002 |
| *Picea sitchensis* | ABK23362 | ACN39834 |
| *Physcomitrella patens* |  | XP_001751217 |
| *Selaginella moellendoffii* |  | XP_002966221 |

Sequences for *Brassica rapa* paralogues Bra001073, Bra001074, Bra032012, Bra032013 were obtained via EnsemblPlants (www.plants.ensembl.org)
