# Supplementary material for: Bidirectional promoters in seed development and related hormone/stress responses
Source: BMC Plant Biol. 2013 Nov 22;13:187. doi: 10.1186/1471-2229-13-187 (PMC4222868; doi:10.1186/1471-2229-13-187)
Supplement: Additional file 3: Table S2 — Sequences of all primers used in the present study. [file 1471-2229-13-187-S3.docx]

| primer | sequence |
| --- | --- |
| 150F | 5'ATGGCAACCACCAGATCGAAG3' |
| 150R | 5'AACATGGTGAGTCTTGTCCG3' |
| 160F | 5’TGAAGCGATTATGGTGGTG3’ |
| 160R | 5’ATGCTCGATCTTGACAACC3’ |
| 190F | 5’ATGGCTCTCGAATGGGTTG3’ |
| 190R | 5’AAAGGACCTGAGGAAGACGAG3’ |
| 165F | 5’TCGCATATGGTCTCTTCACG3’ |
| 165R | 5’AACCAGGCTTTCTCCTCCAT3’ |
| Actin2F | 5’TATCGCTGACCGTATGAGCAAAG5’ |
| Actin2R  150 qF  150qR  160 qF  160qR  18S F  18S R | 5’TGGACCTGCCTCATCATACTCG3’  5’TCTGCTCTCTTCCCTTCACG3’  5’TAGAGGGACCGAAGACTCCT3’  5’GGTGCTATTGACGATGCCAG3’  5’TGGTGACGAAGATGCTCTGA3’  5’ CGGCTACCACATCCAAGGAA3’  5’ TGTCACTACCTCCCCGTGTCA3’ |
| AtpromF | 5’TATCACGGATCAAGCTAGA3’ |
| AtpromR | 5’AAAACTGTGTTTCTTCGTCGGA3’ |
| 150promF | 5’AAAAAGCAGGCT**AC**TATCACCGGATCAAGCTAGA3’ |
| 150promR | 5’AGAAAGCTGGGT**C**AAAACTGTGTTTCTTCGTCGGA3’ |
| 160promF | 5’AAAAAGCAGGCT**AC**AAAACTGTGTTTCTTCGTCGGA3’ |
| 160promR | 5’AGAAAGCTGGGT**C**TATCACCGGATCAAGCTAGA3’ |
| 507L | 5’GGCTAATCCATTTCATGGGAA3’ |
| 507R | 5’CCATCGCACGAAGGCATAGTT3’ |
| 262L | 5’CAAAAGGGTTCCCTGATTACC3’ |
| 262R | 5’ATCTGATCCACGTGTCTCACC3’ |
| LBb1.3 | 5’ATTTTGCCGATTTCGGAAC 3’ |

**Additional file 3: Table S2. Sequence of all primers used in the present study.**
