# Supplementary material for: Bidirectional promoters in seed development and related hormone/stress responses
Source: BMC Plant Biol. 2013 Nov 22;13:187. doi: 10.1186/1471-2229-13-187 (PMC4222868; doi:10.1186/1471-2229-13-187)
Supplement: Additional file 4: Table S3 — Transmission efficiency through the male and female gametophyte in SALK_121507. [file 1471-2229-13-187-S4.docx]

**Additional file 4: Table S3. Transmission efficiency through the male and female gametophyte in SALK_121507**

| **Cross** | **No of F1 seedlings** | **T-DNA insertion** | **No T-DNA insertion** | **TE (%)** |
| --- | --- | --- | --- | --- |
| Col-0 x SALK_121507(het) | 75 | 31 | 44 | 70.5 |
| SALK_121507(het)x Col-0 | 47 | 22 | 25 | 88.0 |
